# FoxO1a mediated cadmium‐induced annulus fibrosus cells apoptosis contributes to intervertebral disc degeneration in smoking

**DOI:** 10.1002/jcp.29895

**Published:** 2020-07-11

**Authors:** Doudou Jing, Wei Wu, Xiangyu Deng, Yizhong Peng, Wenbo Yang, Donghua Huang, Zengwu Shao, Dong Zheng

**Affiliations:** ^1^ Department of Orthopaedics, Union Hospital, Tongji Medical College Huazhong University of Science and Technology Wuhan China; ^2^ Department of Orthopedics, Musculoskeletal Tumor Center The Second Affiliated Hospital of Zhejiang University School of Medicine Hangzhou China

**Keywords:** annulus fibrosus cells, apoptosis, cadmium, FoxO1a, intervertebral disc degeneration

## Abstract

Cadmium (Cd), a type of heavy metal that accumulates in the body because of smoking, mediates the toxic effect of smoking in many diseases, such as cardiovascular disease, osteoarthritis, and osteoporosis. However, the toxic effect of Cd on intervertebral disc tissues have not been reported. In the current study, we demonstrated that Cd induced the apoptosis of annulus fibrosus (AF) cells, which contributed to intervertebral disc degeneration (IVDD). Specifically, Cd induced the nuclear translocation of FoxO1a, which drives AF cells apoptosis through mitochondrial‐related pathway. Phosphatidylinositol 3‐kinase/protein kinase B (PI3K/AKT) signal pathway is also involved in this process. The combined use of LY29002, an inhibitor of PI3K, and small interfering RNA‐targeting FoxO1a confirmed the relationship between the PI3K/AKT signal pathway and FoxO1a. In summary, present research explores the mechanism behind the contribution of smoking to IVDD and finds a new feasible target for preventing IVDD in smoking.

## Graphical abstract

Cadmium (Cd), a type of heavy metal that accumulates in the body because of smoking, mediates the occurrence of intervertebral disc (IVD) degeneration. Specifically, Cd induced the nuclear translocation of FoxO1a, which drives annulus fibrosus (AF) cells apoptosis through mitochondrial related pathway. It was the first time to explore the mechanism behind the contribution of smoking to IVD first and finds a feasible target for preventing IVD in smoking.

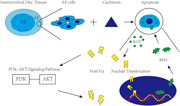

## 1 INTRODUCTION

Low back pain (LBP) seriously affects the quality of life of human beings. More than 70–85% of adults suffer from LBP during their lifetime (Andersson, 1999). The excessive morbidity of LBP has resulted in very large economic burdens to countries and individuals. Lumbar intervertebral disc degeneration (IVDD) is considered to be strongly associated with chronic LBP (Livshits et al., 2011). Intervertebral disc tissue includes the nucleus pulposus and the annulus fibrosus (AF), and the structural stability of intervertebral disc tissue is important for the prevention of IVDD. In fact, well before disc degeneration, the structural stability of the AF is destroyed, which is one of important mechanisms that promote the occurrence of IVDD. The apoptosis of AF cells is an important cause of AF structural destabilization (Hu et al., 2019).

One common cause of IVDD is smoking (Elmasry, Asfour, de Rivero Vaccari, & Travascio, 2015; Maher, Underwood, & Buchbinder, 2017). Tobacco‐derived nicotine has been reported to be highly correlated with IVDD by affecting the GAG concentration at the cartilage endplate and nucleus pulposus (Elmasry et al., 2015). However, it is difficult to fully explain the negative effect of smoking on intervertebral disc. Cadmium (Cd), a type of heavy metal, accumulates in the body due to smoking, which has a toxic effect on many organs and tissues and causes multiple diseases, including cardiovascular diseases, osteoporosis, and lung cancer (Bernhard, Rossmann, & Wick, 2005; Cosselman, Navas‐Acien, & Kaufman, 2015; Filippini et al., 2016; Galazyn‐Sidorczuk, Brzoska, & Moniuszko‐Jakoniuk, 2008; Nawrot et al., 2015; Pi et al., 2019). Whether Cd contributes to IVDD is unknown. The results of the current study are the first to demonstrate that Cd induces AF cell apoptosis through the mitochondrial apoptosis pathway. The proapoptosis effect of reactive oxygen species (ROS) and FoxO1a was involved in the occurrence of AF cell apoptosis after Cd treatment. Moreover, the phosphatidylinositol 3‐kinase/protein kinase B (PI3K/AKT) signal pathway played a key role in reducing AF cell apoptosis. This study explored mechanism behind the contribution of smoking to IVDD and found a feasible target for preventing IVDD in smoking.

## 2 MATERIALS AND METHODS

### 2.1 Antibodies and reagents

Cadmium chloride (439800) was purchased from Sigma‐Aldrich. Rapamycin (S1039) and 3‐methyladenine (S2767) were purchased from Selleck. Bad A1 (A8510; China) was purchased from Solarbio. LY294002 (HY‐10108; China) was purchased from MedChemExpress.

### 2.2 Cell culture

All the experiments were approved by the Animal Care and Ethics Committee of Huazhong University of Science and Technology in this study. Rat AF cells were harvested from intervertebral disc tissues of Sprague–Dawley rats (240–260 g). Ten male Sprague–Dawley rats were killed by an overdose of sodium pentobarbital. The intervertebral disc tissues were collected from the T10 to L5 of rats under aseptic conditions. Then, we separated the AF tissues from the intervertebral disc tissues by using a dissecting microscope. Afterward, AF tissues were treated with 0.25% collagenase I for 8–10 hr at 37°C. Next, the digested tissues were plated on α‐minimum essential medium (α‐MEM; HyClone) with 10% foetal bovine serum (Gibco) and antibiotics (1% penicillin/streptomycin). After culturing in the incubator at 5% CO_2_ and 37°C for 5 days, tissue fragments and suspended cells were removed. The adherent cells were AF cells. When confluence was 80–90%, the cells were collected by using 0.25% trypsin‐ethylenediaminetetraacetic acid (Solarbio) and replated on cell culture plates for subculture. The medium was changed every other day. Third passage cells were used for subsequent experiments.

### 2.3 Treatment protocols and treatment groups

To evaluate the dose‐dependent and time‐dependent effects of Cd, AF cells were exposed to 0, 0.01, 0.05, 0.1, 0.5, 1, 5 μmol/L, of 10 μmol/L Cd for 6, 12, or 24 hr. Phosphate‐buffered saline (PBS) served as a control because it was the vehicle used to dissolve Cd. A Cd concentration of 0, 1, and 5 μmol/L was used for the next experiment, and the length of the Cd treatment was 24 hr.

### 2.4 Cell viability assay

Cell viability was detected by Cell Counting Kit‐8 (CCK‐8), according to the manufacturer's protocol. AF cells were plated on 96‐well plates (5,000 cells per well) and incubated in complete medium for 24 hr. Then, the cells were treated with different Cd concentrations for different treatment times. Then, 100 μl of α**‐**MEM containing 10 μl of CCK‐8 solution was added to each well. After incubation for another 2 hr, the absorbance of the wells was measured by a microplate reader at 450 nm.

### 2.5 Annexin V–propidium iodide staining

An Annexin V‐FITC Apoptosis Detection Kit (C1062M; Beyotime, China) was used to detect the percentage of propidium iodide (PI)‐positive AF cells after Cd treatment. According to the instruction manual, the cells were harvested and resuspended with 200 μl of Annexin V‐FITC binding buffer, and then 5 μl of Annexin V and 5 μl PI were added to the samples. The samples were detected by flow cytometry (Cytoflex; Beckman Coulter). These results were analyzed by CellQuest analysis software (BD).

### 2.6 JC‐1 staining

Mitochondrial membrane potential (MMP) was detected with a JC‐1 Staining Kit (C2006; Beyotime). Cells from different groups were washed with PBS before incubation with a 1.5 ml mixture of JC‐1 staining fluid in the dark for 30 min. Then, the cells were washed with cold staining buffer. Afterward, the MMP of different samples was detected by flow cytometry or fluorescence microscopy (Olympus, Japan). The ratio of red fluorescence intensity to green fluorescence intensity was analyzed with CellQuest analysis software (BD) or ImageJ software.

### 2.7 2′‐7′Dichlorofluorescin diacetate staining assay

The intracellular total ROS level was measured by 2′‐7′dichlorofluorescin diacetate (DCFH‐DA; S0033; Beyotime). After collecting the cells from different samples, the cells were washed twice with PBS and incubated with 10 μmol/L DCFH‐DA in the dark for 20 min. Then, the cells were washed with α‐MEM three times, and the samples were detected by flow cytometry. These results were analyzed by CellQuest analysis software (BD).

### 2.8 Immunofluorescence

Before fixing in 4% paraformaldehyde for 30 min, the samples were washed with PBS three times. Afterward, the samples were washed with PBS an additional three times and treated with 0.5% Triton X‐100 for 20 min. Then, the samples were blocked for 30 min in 5% goat serum albumin. Then, the samples were incubated with anti‐FoxO1a, anti‐FoxO3, and anti‐FoxO4 antibodies (ab52857, ab109629, and ab128908, respectively; Abcam) overnight at 4°C. After washing with PBST, the samples were incubated with a fluorophore‐conjugated secondary antibody for 1 hr in the dark. Then, 4′,6‐diamidino‐2‐phenylindole was used to counterstain the samples in the dark for 5 min. These samples were observed and photographed with a fluorescence microscope (Olympus, Japan).

### 2.9 RNA interference

The AF cells were transfected with either 2 ng FoxO1a‐targeted small small interfering RNA (siRNA) or a control nonspecific siRNA. After transfection, the cells were treated with 1 μmol Cd for 24 hr. Then, the cells were collected and processed for the next experiment.

### 2.10 Real‐time polymerase chain reaction

Total RNA was extracted by using RNA extraction reagent (Takara). According to the manufacturer's protocols, 1 μg of total RNA was transcribed in a 20 μl reaction mixture by using the PrimeScript RT Reagent Kit with gDNA Eraser (Perfect Real Time; Takara) for complementary DNA synthesis. The resulting reverse transcription product was expanded by using the NovoStart® SYBR qPCR SuperMix (Novoprotein, E090‐01A), and the data were collected using an iQ5 Real‐Time PCR Detection System (Bio‐Rad) system. The $2^{-\Delta \Delta C_{t}}$ method was used to analyze the data, and the housekeeping gene glyceraldehyde 3‐phosphate dehydrogenase (GAPDH) was used to normalize the level of messenger RNA. The primer sequences were as follows: GAPDH forward, 5′‐AGGTCGGTGTGAACGGATTTG‐3′; GAPDH reverse, 5′‐TGTAGACCATGTAGTTGAGGTCA‐3′ and Foxo1 forward, 5′‐CCCAGGCCGGAGTTTAACC‐3′; and Foxo1 reverse, 5′‐GTTGCTCATAAAGTCGGTGCT‐3′.

### 2.11 Western blot analysis

After treatment, the total protein of AF cells from different samples was extracted by using a Western and IP Cell Lysis Kit (Beyotime). Protein concentrations were measured by a BCA Protein Assay Kit (Beyotime). Equal aliquots of protein from each sample were separated by 12% sodium dodecyl sulfate‐polyacrylamide gel electrophoresis and subsequently transferred to polyvinylidene difluoride membranes (Millipore). Then, the membranes were blocked by 5% nonfat milk in TBST buffer and incubated overnight at 4°C with primary antibodies, including GAPDH (1:5,000, ab9485; Abcam), Bax (ab32503, 1:1,000; Abcam), Bcl‐2 (ab32124, 1:1,000; Abcam), cytochrome c (Cyt c; ab13575, 1:1,000; Abcam), FoxO1a (ab52857, 1:1,000; Abcam), FoxO3 (ab109629, 1:1,000; Abcam), FoxO4 (ab128908, 1:1,000; Abcam), p‐FoxO1a (S256; ab131339, 1:1,000; Abcam), p‐FoxO1a (S319; ab47328, 1:1,000; Abcam), BIM (ab32158, 1:1,000; Abcam), PI3K (ab151549, 1:1,000; Abcam), AKT (ab170463, 1:1,000; Abcam), and phospho‐AKT (p‐AKT; ab81283, 1:1,000; Abcam). Afterwards, the membranes were incubated with secondary antibodies for 1 hr at room temperature. After washing with PBS three times, the bands were observed in a darkroom and quantified using the ImageJ software.

### 2.12 Statistical analysis

All data are presented as the mean ± standard deviation from at least three independent experiments. Student's t test or one‐way analysis of variance was performed to conduct statistical analysis by using GraphPad Prism. *p* < .05 was considered statistically significant.

## 3 RESULTS

### 3.1 Cd induces AF cell apoptosis through the mitochondrial pathway

**Figure 1:**
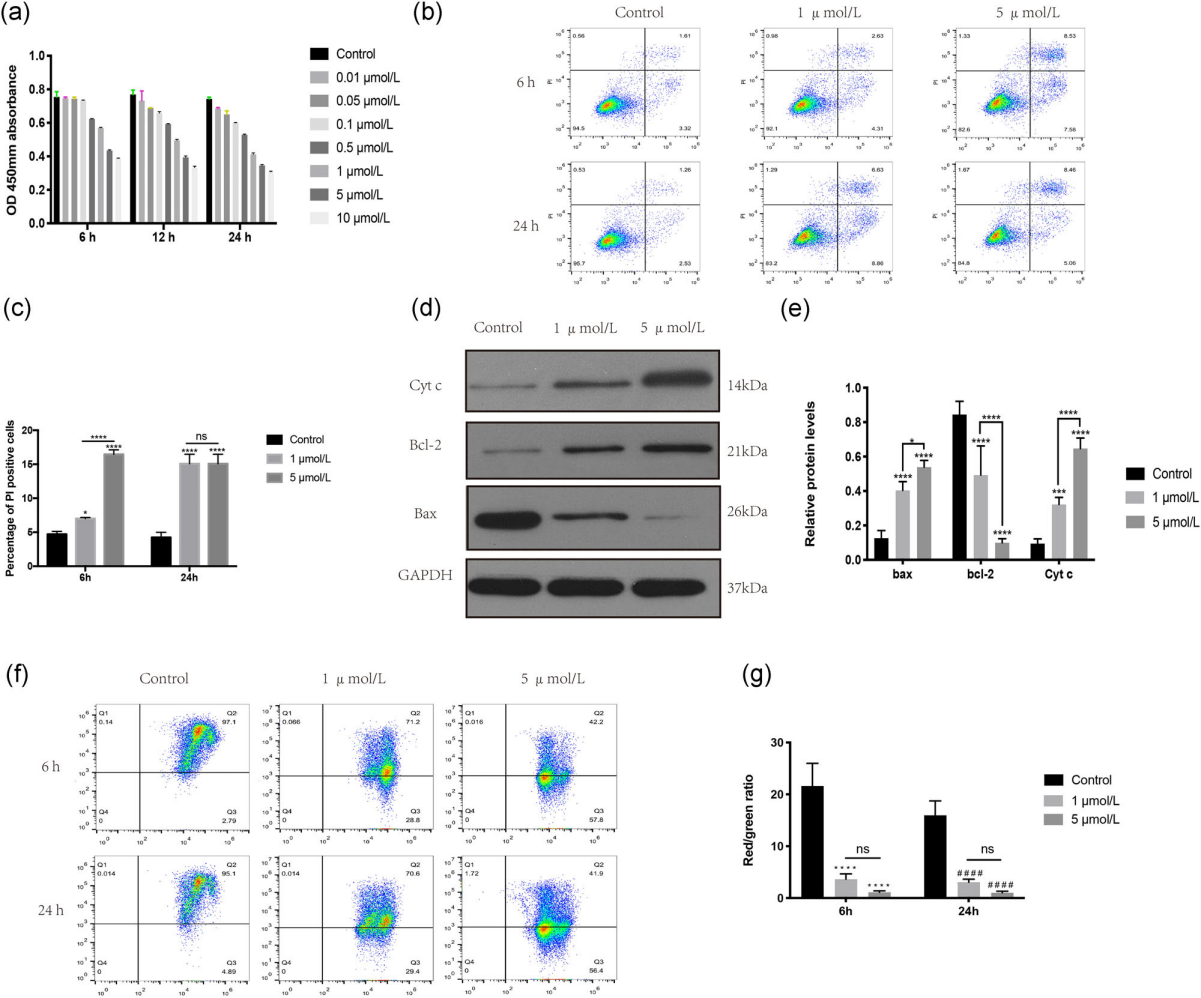
Cd induces the apoptosis of AF cells through the mitochondrial pathway. (a) CCK‐8 assay was used to detect cell viability. To determine the dose‐dependent and time‐dependent effects of Cd on AF cells, cells were treated with different concentrations of Cd for 6, 12, and 24 hr. PBS was used as a control. (b) These figures were obtained from the results of flow cytometry analysis. Annexin V/PI staining was used to detect the percentage of dead cells after treatment with different Cd concentrations for 6 or 24 hr. Annexin−/PI− represents live cells, and Annexin+/PI+ and Annexin+/PI− represent dead cells. (c) Histogram showing the percentage of PI‐positive AF cells from the different groups mentioned in (b). The results from three independent experiments are presented as the mean ± *SD* (*p < .05, **p < .01, ***p < .001, ****p < .0001). (d) After exposure to 1 μmol/L Cd for 24 hr, the expression levels of Cyt c, Bcl‐2, Bax, and GAPDH were determined by western blot analysis. (e) Histogram showing the relative protein levels of Cyt c, Bax, and Bcl‐2. The results of three independent experiments are presented as the mean ± *SD* (*p < .05, **p < .01, ***p < .001, ****p < .0001). (f) These graphs were obtained from flow cytometry analysis. After treatment with different Cd concentrations for 6 or 24 hr, JC‐1 staining was used to detect the MMP of AF cells. (g) Histogram showing the red fluorescence/green fluorescence intensity ratio of different samples. The results of three independent experiments are presented as the mean ± *SD* (*^/#^ *p* ^^< .05, ^##/^**p < .01, ^###/^***p < .001, ^####/^****p < .0001). AF, annulus fibrosus; CCK‐8, Cell Counting Kit‐8; Cd, cadmium; Cyt c, cytochrome c; GAPDH, glyceraldehyde 3‐phosphate dehydrogenase; *ns*, no statistically significant difference; PBS, phosphate‐buffered saline; OD, optical density; PI, propidium iodide; *SD*, standard deviation

In CCK‐8 assays, the viability of AF cells was decreased with increasing Cd concentration and treatment duration, as shown by the decrease in the optical density at 450 mm (Figure 1a). Next, we used an Annexin V‐FITC Apoptosis Detection Kit to evaluate the apoptosis of AF cells under different Cd concentrations and different treatment durations. The results showed that the apoptosis of AF cells increased with increasing Cd concentrations (Figure 1b,c). Afterward, we used a western blot assay to further explore the causes of AF cell apoptosis. As shown in Figure 1d,e, proteins related to the mitochondrial apoptosis pathway were altered. The expression of Bax was upregulated with increasing Cd concentration, while the expression of Bcl‐2 was downregulated. In addition, Cyt c, a biomarker of mitochondrial‐related apoptosis, was also upregulated. These results indicated that the mitochondrial pathway was involved in Cd‐induced AF cell apoptosis. Many reports have stated that decreased MMP is a landmark event in the early stage of mitochondrial‐related cell apoptosis (Vringer & Tait, 2019). To further explore the role that mitochondria play in Cd‐induced AF cell apoptosis, we measured the MMP of AF cells by performing JC‐1 staining and flow cytometry analysis. As shown in Figure 1f,g, Cd induced the loss of MMP in a dose‐dependent manner, as shown by the decrease in the ratio of red fluorescence (the state of aggregates) to green fluorescence (the state of monomers).

### 3.2 Cd‐induced AF cell apoptosis was related to excessive intracellular ROS

**Figure 2:**
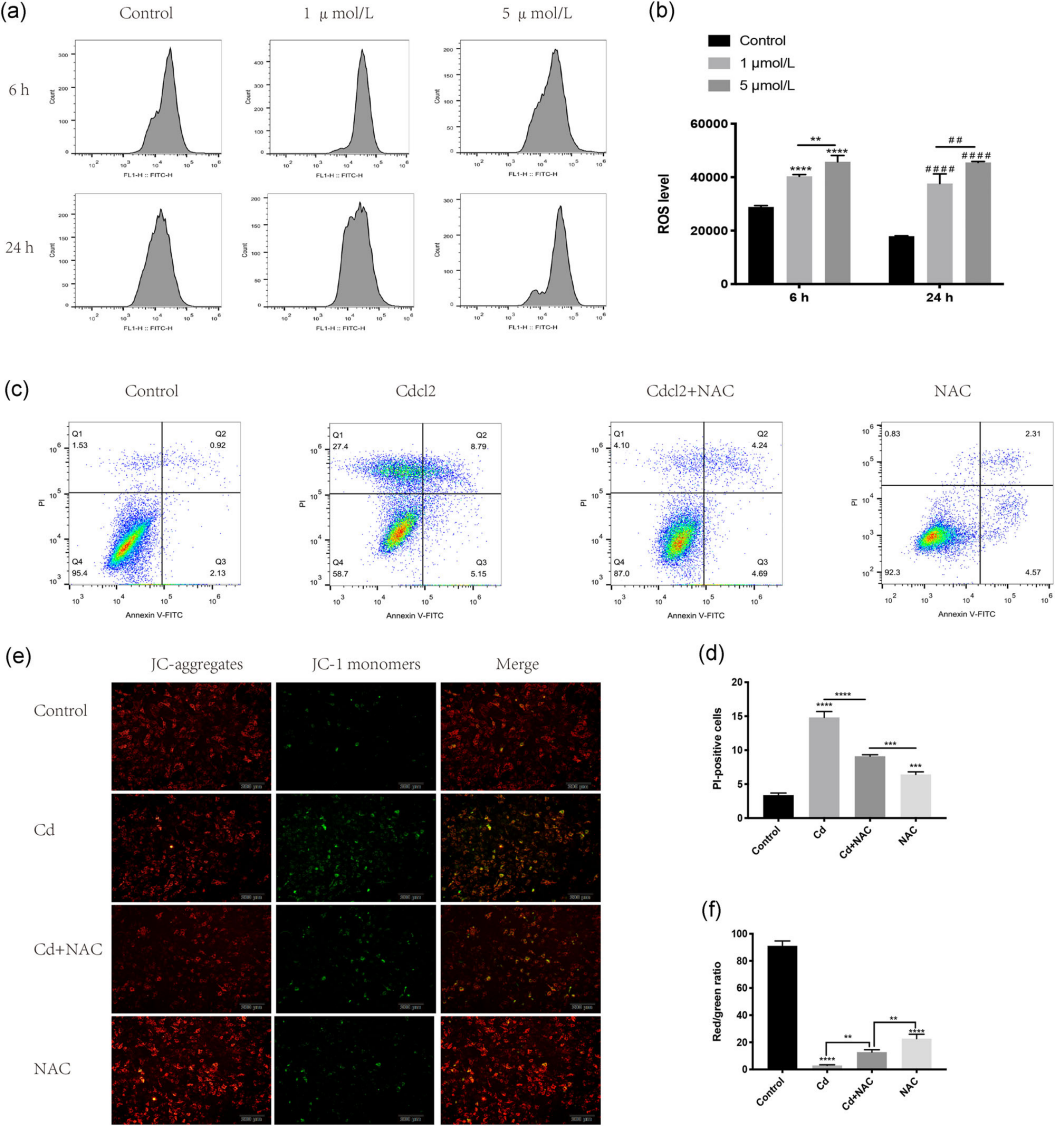
Cd‐induced AF cell apoptosis was related to excessive intracellular ROS. (a) These graphs were obtained from flow cytometry analysis. After treatment with different Cd concentrations for 6 or 24 hr, we used DCFH‐DA fluorescent probes to evaluate ROS levels in AF cells. (b) Histogram showing intracellular ROS levels from different samples. The results of three independent experiments are presented as the mean ± *SD* (*^/#^ *p* < .05, ^##/^**p < .01, ^###/^***p < .001, ^####/^****p < .0001). (c) These graphs were obtained from flow cytometry analysis. Before Cd treatment, NAC (5 mmol/L) was used to pretreat AF cells for 1 hr. Annexin V/PI staining was used to detect the percentage of dead cells. (d) Histogram showing the percentage of PI‐positive AF cells from different groups. The results of three independent experiments are presented as the mean ± *SD* (*p < .05, **p < .01, ***p < .001, ****p < .0001). (e) A JC‐1 Staining Kit was used to detect the MMP of AF cells, which was observed and visualized by fluorescence microscopy. (f) After analysis and measurement by the ImageJ software, the histogram shows the fluorescence intensity ratio of red to green from different groups. The results of three independent experiments are presented as the mean ± *SD* (*p < .05, **p < .01, ***p < .001, ****p < .0001). AF, annulus fibrosus; Cd, cadmium; DCFH‐DA, 2′‐7′dichlorofluorescin diacetate; MMP, mitochondrial membrane potential; NAC, *N*‐acetyl‐l‐cysteine; *ns*, no statistically significant difference; PI, propidium iodide; ROS, reactive oxygen species; *SD*, standard deviation

An excessively high level of intracellular ROS could induce the opening of a high conductance pore in the inner mitochondrial membrane, leading to the release of Cyt c from the mitochondria to the cytoplasm and activating the mitochondrial apoptosis pathway (Vakifahmetoglu‐Norberg, Ouchida, & Norberg, 2017). To further explore whether ROS are involved in Cd‐induced AF cell apoptosis, we used DCFH‐DA fluorescent probes to evaluate ROS levels in AF cells. As shown in Figure 2a,b, intracellular ROS levels increased in a dose‐dependent and time‐dependent manner. Then, we used 5 mmol/L *N*‐acetyl‐l‐cysteine (NAC), an antioxidant, to verify the role of ROS in the AF cell apoptosis induced by Cd. The percentage of AF cell apoptosis was decreased by pretreating the AF cells with NAC for 1 hr before Cd treatment (Figure 2c,d). The MMP of AF cells was also increased after pretreatment with NAC, as indicated by the increased ratio of red fluorescence to green fluorescence. Collectively, these results confirmed that excessive intracellular ROS play a key role in Cd‐induced AF cell apoptosis.

### 3.3 FoxO1a was upregulated and translocated to the nucleus from the cytoplasm after Cd treatment

**Figure 3:**
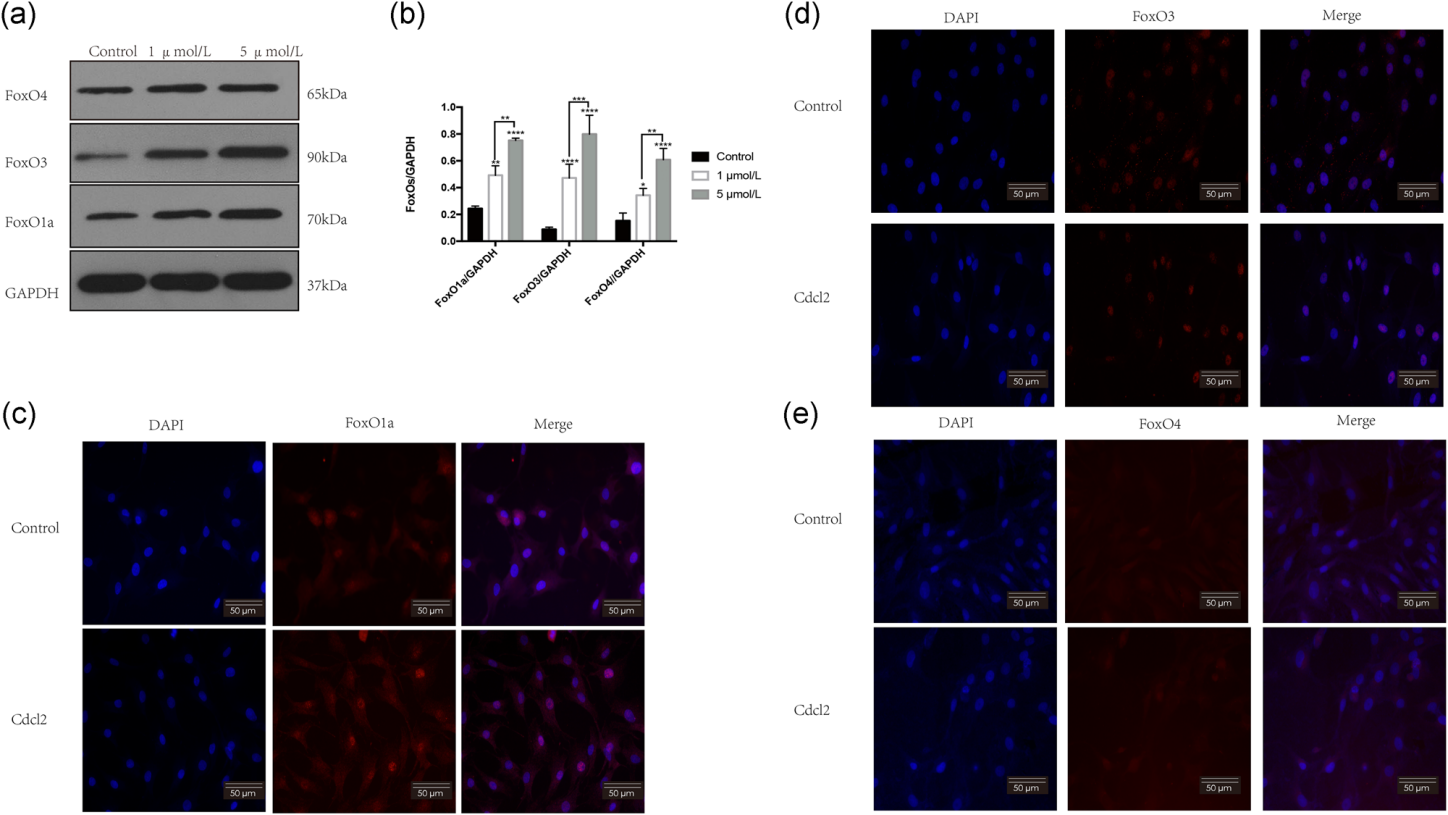
FoxO1a was involved in AF cell apoptosis following Cd treatment. (a) After exposure to Cd for 24 hr, the expression of FoxO1a, FoxO3, and FoxO4 was determined by western blot analysis. (b) Histogram showing the relative protein levels of FoxO1a, FoxO3, and FoxO4. The results of three independent experiments are presented as mean ± *SD* (*p < .05, **p < .01, ***p < .001, ****p < .0001). (c–e) The subcellular location of FoxO1a, FoxO3, and FoxO4 was detected by immunofluorescence staining. AF, annulus fibrosus; Cd, cadmium; DAPI, 4′,6‐diamidino‐2‐phenylindole; GAPDH, glyceraldehyde 3‐phosphate dehydrogenase; *SD*, standard deviation

Many reports stated that forkhead‐box transcription factors play a vital role in the maintenance of mitochondrial function by mediating the expression of multiple genes related to mitochondrial antioxidant enzymes and apoptosis (Kim & Koh, 2017; Zhang, Tang, Hadden, & Rishi, 2011). On the basis of these reports, we investigated whether the FoxO family was involved in Cd‐induced AF cell apoptosis. We detected the expression of FoxO1a, FoxO3, and FoxO4 by western blot analysis. As shown in Figure 3a,b, the expression of these proteins was upregulated with increasing Cd concentrations. In fact, the functional activation of FoxOs was dependent on translocation from the cytoplasm into the cell nucleus. Therefore, we detected the subcellular location of FoxOs by using immunofluorescence staining. We observed that only FoxO1a was translocated into the cell nucleus after Cd treatment (Figure 3c–e). These results suggested that FoxO1a was involved in Cd‐induced AF cell apoptosis.

### 3.4 The AF cell apoptosis was rescued by knocking down FoxO1a

**Figure 4:**
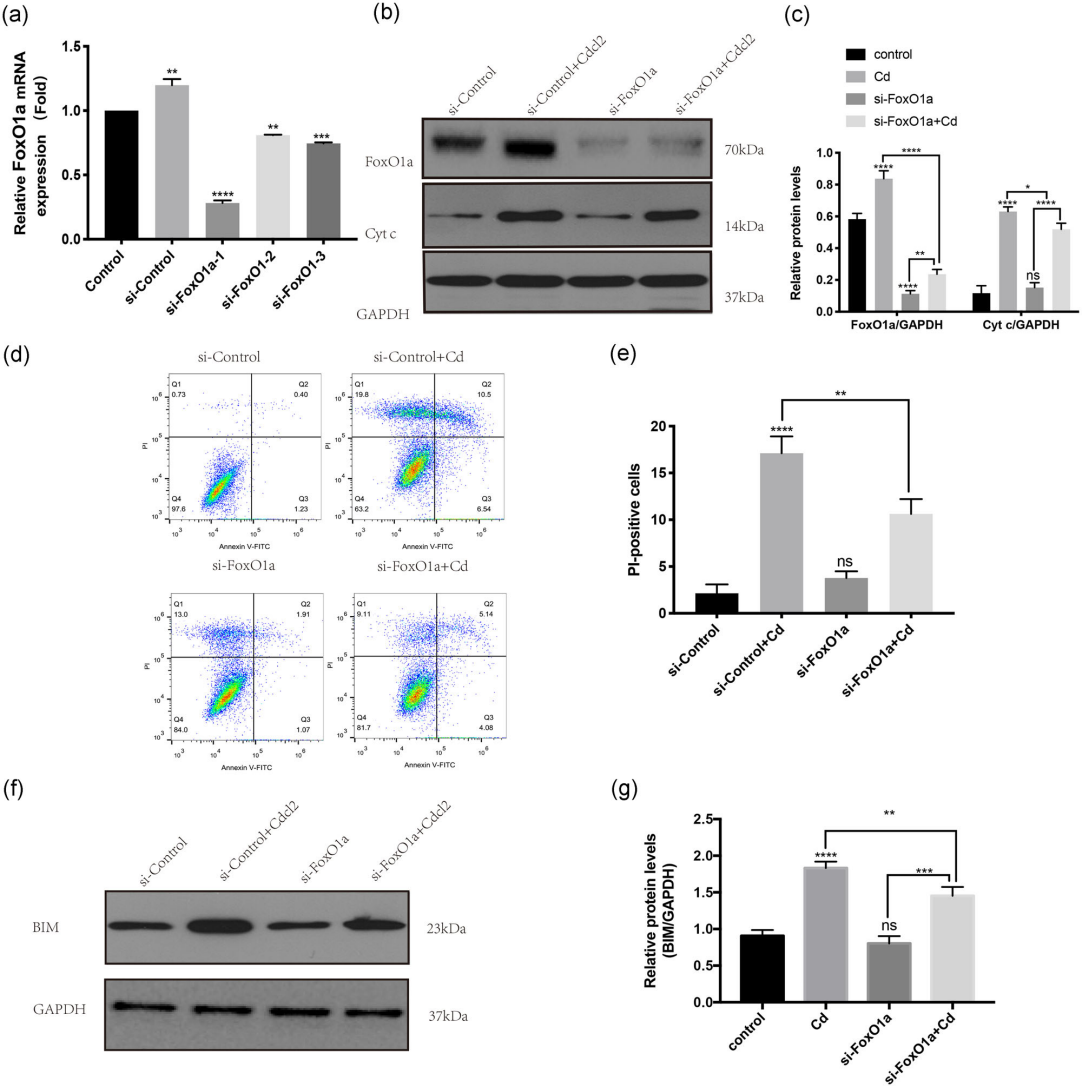
AF cell apoptosis was rescued by FoxO1a knockdown. (a) Real‐time PCR was used to confirm the efficiency of FoxO1a knockdown. Histogram analysis showed the relative mRNA levels of FoxO1a. The results of three independent experiments are presented as mean ± *SD* (*p < .05, **p < .01, ***p < .001, ****p < .0001). (b) Western blot analysis was used to detect FoxO1a, Cyt c, and GAPDH in samples treated with different concentrations of FoxO1a. (c) Histogram analysis showing the relative protein levels of FoxO1a and Cyt c. The results of three independent experiments are presented as mean ± *SD* (*p < .05, **p < .01, ***p < .001, ****p < .0001). (d) These figures were obtained from the results of flow cytometry analysis. Annexin V/PI staining was used to detect the percentage of dead cells in different treatment groups. (e) Histogram analysis showing the percentage of PI‐positive cells. The results of three independent experiments are presented as mean ± *SD* (*p < .05, **p < .01, ***p < .001, ****p < .0001). (f) Western blot analysis was used to detect BIM and GAPDH in samples from different treatment groups. (g) Histogram analysis showed the relative protein levels of BIM. The results of three independent experiments are presented as mean ± *SD* (*p < .05, **p < .01, ***p < .001, ****p < .0001). AF, annulus fibrosus; Cd, cadmium; Cyt c, cytochrome c; GAPDH, glyceraldehyde 3‐phosphate dehydrogenase; mRNA, messenger RNA; PCR, polymerase chain reaction; PI, propidium iodide; *SD*, standard deviation

On the basis of above results, we used siRNA to knockdown FoxO1a expression in AF cells to further explore the role of FoxO1a in AF cell apoptosis. The knockdown efficiency was confirmed by real‐time PCR (Figure 4a). As shown in Figure 4b,c, Cyt c, a protein related to apoptosis, was upregulated after Cd treatment. However, the expression of Cyt c was downregulated in the group with siRNA‐mediated FoxO1a knockdown before Cd treatment compared to the Cd and si‐control treatment group. Flow cytometry results also showed that the knockdown of FoxO1a could reduce the percentage of dead cells following Cd treatment (as shown in Figure 4d,e). In addition to these changes, BIM, a target gene of FoxO1a that promotes apoptosis, was upregulated after Cd treatment. FoxO1a knockdown reduced the expression level of BIM (shown in Figure 4f,g). These results indicated that the AF cell apoptosis caused by Cd was rescued by FoxO1a knockdown.

### 3.5 The PI3K/AKT/FoxO1a signal pathway is involved in Cd‐induced AF cell apoptosis

**Figure 5:**
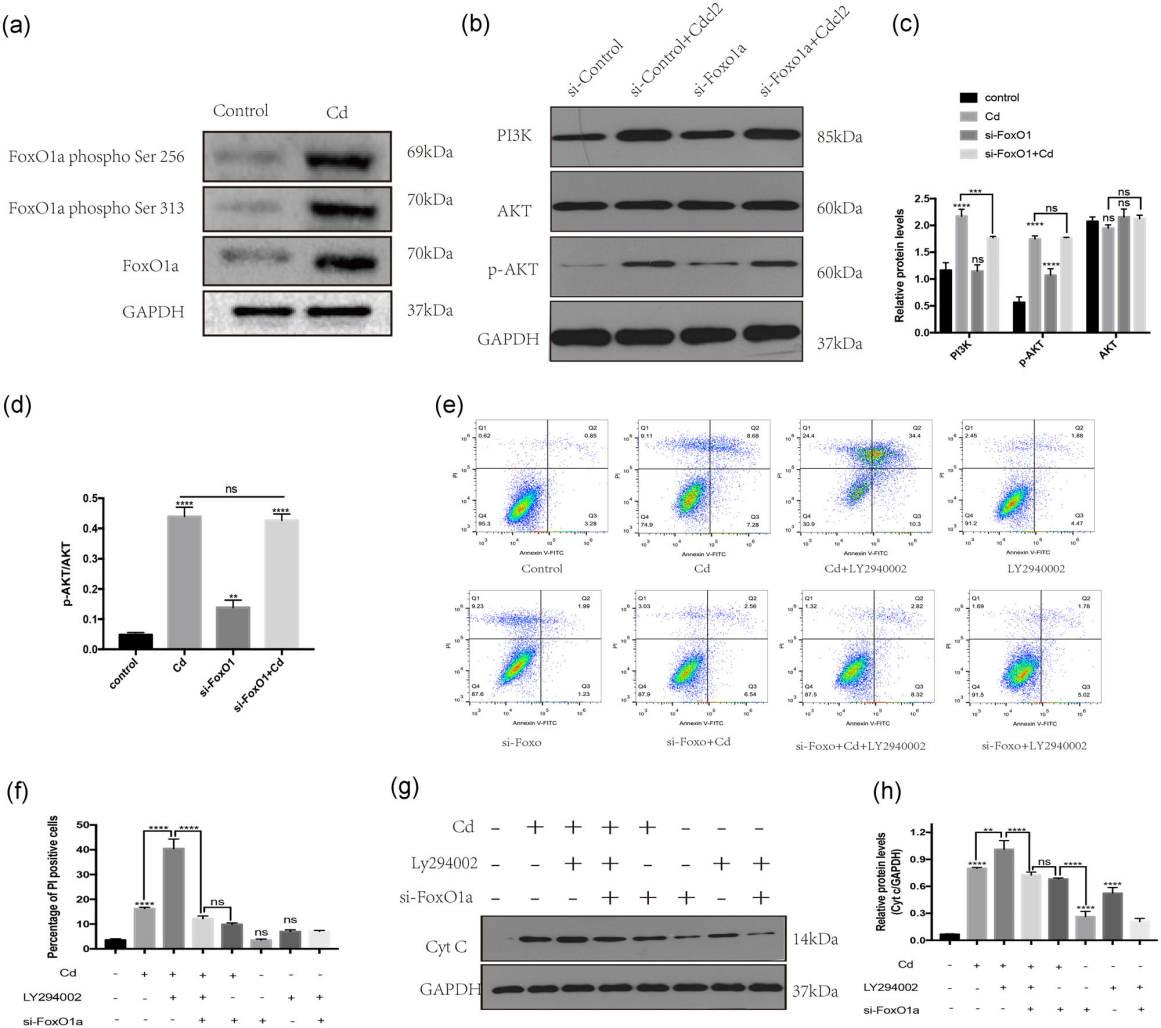
The PI3K/AKT/FoxO1a signal pathway is involved in Cd‐induced AF cell apoptosis. (a) Western blot analysis was used to detect the expression of FoxO1a, the phosphorylation of FoxO1a at Ser 256 and Ser 313, and the GAPDH level in samples with different treatments. (b) Western blot analysis was used to detect the expression of PI3K, AKT, p‐AKT, and GAPDH in samples from different treatment groups. (c) Histogram analysis showing the relative protein levels of PI3K, AKT, and p‐AKT. The results of three independent experiments are presented as mean ± *SD* (*p  < .05, **p < .01, ***p < .001, ****p < .0001). (d) Histogram analysis showed the ratio of protein levels of p‐AKT to AKT. The results of three independent experiments are presented as mean ± *SD* (*p < .05, **p < .01, ***p < .001, ****p < .0001). (e) These figures were obtained from the results of flow cytometry analysis. Annexin V/PI staining was used to detect the percentage of dead cells from groups with different treatments. (f) Histogram analysis showing the percentage of PI‐positive cells. The results of three independent experiments are presented as mean ± *SD* (*p < .05, **p < .01, ***p < .001, ****p < .0001). (g) Western blot analysis was used to detect the expression of Cyt c and GAPDH in samples from different treatment groups. (h) Histogram analysis showing the relative protein levels of Cyt c. The results of three independent experiments are presented as mean ± *SD* (*p < .05, **p < .01, ***p < .001, ****p < .0001). AF, annulus fibrosus; Cd, cadmium; Cyt c, cytochrome c; GAPDH, glyceraldehyde 3‐phosphate dehydrogenase; p‐AKT, phospho‐AKT; PI, propidium iodide; PI3K/AKT, phosphatidylinositol 3‐kinase/protein kinase B; *SD*, standard deviation

Moreover, FoxOs are also regulated by posttranslational modification, most prominently phosphorylation, which causes FoxO inactivation and nuclear exclusion (Hamann & Klotz, 2013). As shown in Figure 5a, the phosphorylation of FoxO1a at Ser 256 and Ser 313 was upregulated with Cd treatment. However, the kinase that mediates these processes is still unknown. The PI3K/Akt signal pathway regulates cell survival and apoptosis by phosphorylating various proteins. To determine whether the PI3K/Akt signal pathway is involved in the process of Cd‐induced cell apoptosis, we detected the key proteins in the PI3K/Akt signal pathway. As shown in Figure 5b–d, the western blot results showed that the expression of PI3K and the ratio of p‐AKT to AKT were upregulated following Cd treatment, which indicated that the PI3K/AKT pathway was activated. Then, to further explore the link among the PI3K/Akt signal pathway, FoxO1a and Cd‐induced cell apoptosis, we used a rescue experiment to confirm their relationships. As shown in Figure 5e,f, pretreatment with LY294002 (an inhibitor of PI3K) in AF cells increased the percentage of AF cell apoptosis following Cd treatment. However, compared to Cd and LY294002 treatment alone, knocking down FoxO1a in AF cells before Cd and LY294002 treatment could rescue AF cell apoptosis. These results were also confirmed by detecting the expression of Cyt c with western blot analysis (shown in Figure 5g,h).

## 4 DISCUSSION

Smoking affects human health and causes kinds of disease, such as cerebrovascular disease (C. Chen et al., 2018), cardiovascular disease (Navas‐Acien et al., 2004), osteoporosis (X. Chen, Zhu, Jin, & Gu, 2009), and IVDD (Livshits, Cohen, Higla, & Yakovenko, 2001). Many chemicals derived from tobacco may be involved in these processes according to many studies, such as nicotine (Elmasry et al., 2015) and Cd. However, the mechanism behind them remains unclear. In the current study, we first found that Cd induces AF cell apoptosis, which may contribute to the occurrence of IVDD by affecting the structural stability of AF. As AF is a vital part of intervertebral disc tissue, the structural stability of AF is important for preventing the degeneration of intervertebral discs. In fact, well before disc degeneration, the structural stability of the AF is destroyed. Although there are no reports regarding Cd concentrations in intervertebral disc tissue, the concentration of Cd in the blood is >30 mg in smokers (Ellis, Vartsky, Zanzi, Cohn, & Yasumura, 1979), which is toxic to many types of cells. We observed that Cd induced AF cell apoptosis through a mitochondrial‐related pathway. The PI3K/AKT/FoxO1a pathway is involved in this process to protect AF cells from apoptosis following Cd treatment. Thus, the results of this study not only establish a link between smoking and IVDD but also provide a feasible therapy to relieve Cd toxicity in intervertebral disc tissue.

Cd bonds to sulphydryl groups of proteins and depletion of glutathione and then induces excessive oxidative stress to show its toxic effects (Jomova & Valko, 2011). Indeed, ROS mediates Cd toxic effect in various cells and tissues. It should be mentioned that mitochondria seems to be a major target of Cd toxic effects. Cristina found that Cd induces mitochondrial dysfunction through generating excessive intracellular ROS in human osteoblasts and results in bone loss. Antioxidant enzymes, including glutathione peroxidase 1, glutathione S‐reductase, and superoxide dismutase (SOD1 and SOD2) also exhibited a trend toward decrease in transcripts after Cd treatment (Monteiro et al., 2018). In addition, ROS mediated abnormal mitochondrial dynamics are also increasingly implicated in Cd stress (Xu et al., 2016). In addition to leading mitochondria dysfunction, excessive intracellular ROS could destabilize others various organelle, like lysosome, leads to the release of organelle contents, such as protease, and result in cell apoptosis (Cai et al., 2018). In addition, as an intracellular signal, ROS also activates the expression of many types of genes directly or indirectly, such as genes related to antioxidant activity or genes related to cell apoptosis (Paul et al., 2014; Pohl, Agostino, Dharmarajan, & Pervaiz, 2018). Collectively, these results suggest that ROS play a key role in Cd‐induced cell apoptosis.

FoxOs, a transcription factor family, activated and translocated to cell nucleus by ROS, regulates cell fate including cell apoptosis and proliferation (Fu & Tindall, 2008; Klotz et al., 2015; Putker et al., 2013). In mammals, the FoxOs family ubiquitously expresses in multiple cell types and has several members, such as FoxO1a, FoxO3, FoxO4, and FoxO6. Abnormal FoxOs functions are related to several diseases, such as osteoarthritis and osteoporosis (Matsuzaki et al., 2018; Schaffner et al., 2018). However, the role of FoxOs in intervertebral disc tissues is rarely reported. Therefore, in the current experiments, we focused the relationship between intracellular ROS and FoxOs after Cd treatment (FoxO6 was not included in this study because FoxO6 is primarily expressed in the brain [Zhang et al., 2011]). As shown in Figures 3, 4, 5, we concluded that FoxO1a played a proapoptosis role in Cd‐induced AF cell apoptosis. The rescued effect brought by FoxO1a knocking down verified it. FoxOs are also regulated by PI3K/AKT signal pathway through posttranslational modification, shown with the upregulation of PI3K and p‐AKT and phosphorylation of FoxO1a at Ser 256 and Ser 313. In addition, rescue experiment with LY294002 and si‐FoxO1a further confirmed that FoxO1a was regulated by the PI3K/AKT signal pathway. Collectively, PI3K/AKT/FoxO1a axis is involved in Cd‐induced AF cells apoptosis. More important, FoxO1a seems like a feasible target to prevent IVDD in smoking.

This study also has some limitations. First, we have not directly evaluated the effect of smoking on intervertebral discs, although there is plenty of evidence that IVDD is related to smoking (Matsuzaki et al., 2018; Schaffner et al., 2018). Second, in the current study, rat AF cells were used to assess the toxic effect of Cd on intervertebral disc tissue because of harvesting limitation of healthy human AF cells. Finally, no experiments were performed in vivo, which limits our understanding of the true effect of smoking and Cd on intervertebral disc tissue. Therefore, further experiments are needed to determine the effects of smoking and Cd on intervertebral disc tissue in vivo.

**Figure 6:**
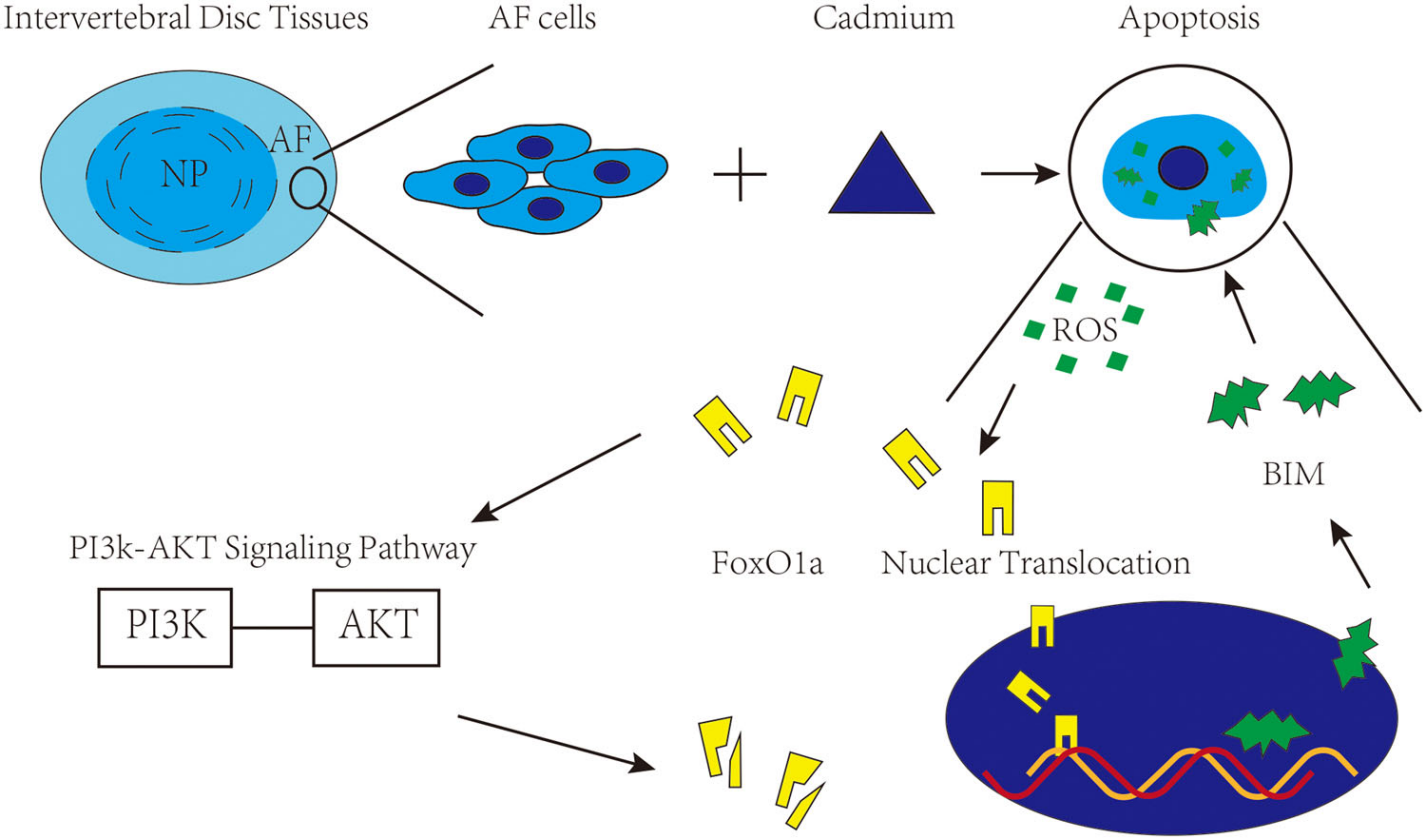
Schematic illustration shows the mechanism involved in cadmium‐induced apoptosis of annulus fibrosus (AF) cells. NP, nucleus pulposus; PI3K/AKT, phosphatidylinositol 3‐kinase/protein kinase B; ROS, reactive oxygen species

In summary, the results of the current study show that Cd induced the apoptosis of AF cells contributes to IVDD, which may mediate the effects of smoking on intervertebral disc tissues. The proapoptosis effect of ROS and FoxO1a induces AF cells apoptosis through the mitochondrial‐related pathway. The PI3K/AKT signal pathway regulates FoxO1a and prevent AF cells from apoptosis (a schematic of the proposed Cd‐induced cell apoptosis mechanism is summarized in Figure 6). Thus, current study explores the mechanism behind the contribution of smoking to IVDD and finds a feasible target for preventing IVDD in smoking.

## CONFLICT OF INTERESTS

The authors declare that there are no conflict of interests.

## AUTHOR CONTRIBUTIONS

D. Z. and Z. S. conceived and designed the study. D. J. and W. W. contributed to carry out the experiments. D. H. and W. Y. contributed to data analysis. D. J. and X. D. wrote the manuscript. Y. P. and Z. S. supervised the research. All authors read and approved the final manuscript.

## Data Availability

The data used to support the findings of this study are available from the corresponding author.
